# Bigmelon: tools for analysing large DNA methylation datasets

**DOI:** 10.1093/bioinformatics/bty713

**Published:** 2018-08-23

**Authors:** Tyler J Gorrie-Stone, Melissa C Smart, Ayden Saffari, Karim Malki, Eilis Hannon, Joe Burrage, Jonathan Mill, Meena Kumari, Leonard C Schalkwyk

**Affiliations:** 1 School of Biological Sciences, University of Essex, Colchester, UK; 2 Institute for Social and Economic Research, University of Essex, Colchester, UK; 3 Department of Psychological Sciences, Birkbeck, University of London, London, UK; 4 Department of Non-Communicable Disease Epidemiology, London School of Hygiene and Tropical Medicine, London, UK; 5 MRC Unit, The Gambia and MRC International Nutrition Group, London School of Hygiene and Tropical Medicine, London, UK; 6 Institute of Psychiatry, Psychology and Neuroscience, King’s College London, London, UK; 7 University of Exeter Medical School, University of Exeter, Exeter, UK

## Abstract

**Motivation:**

The datasets generated by DNA methylation analyses are getting bigger. With the release of the HumanMethylationEPIC micro-array and datasets containing thousands of samples, analyses of these large datasets using R are becoming impractical due to large memory requirements. As a result there is an increasing need for computationally efficient methodologies to perform meaningful analysis on high dimensional data.

**Results:**

Here we introduce the bigmelon R package, which provides a memory efficient workflow that enables users to perform the complex, large scale analyses required in epigenome wide association studies (EWAS) without the need for large RAM. Building on top of the CoreArray Genomic Data Structure file format and libraries packaged in the gdsfmt package, we provide a practical workflow that facilitates the reading-in, preprocessing, quality control and statistical analysis of DNA methylation data.

We demonstrate the capabilities of the bigmelon package using a large dataset consisting of 1193 human blood samples from the Understanding Society: UK Household Longitudinal Study, assayed on the EPIC micro-array platform.

**Availability and implementation:**

The bigmelon package is available on Bioconductor (http://bioconductor.org/packages/bigmelon/). The Understanding Society dataset is available at https://www.understandingsociety.ac.uk/about/health/data upon request.

**Supplementary information:**

Supplementary data are available at *Bioinformatics* online.

## 1 Introduction

DNA methylation is the most easily analyzed, and probably the most stable epigenetic mark. There are multiple site-specific assay methods for DNA methylation based on bisulfite conversion, and currently the most used genome-wide method are micro-arrays made by Illumina, based upon genotyping technology. This has made Epigenome-Wide Association Studies (EWAS) (Rakyan *et al.*, 2011) possible, analogous to genome-wide association studies (GWAS). EWAS have been dominated by the use of the Illumina Infinium HumanMethylation450 BeadChip micro-array, or 450K array (Bibikova *et al.*, 2011), which allows for the interrogation of DNA methylation levels of more than 450 000 loci at a relatively low-cost. The 450K has been used widely and as of July 2017, data from more than 60 000 arrays have been deposited onto the Gene Expression Omnibus (under GPL13534). The 450K has since been superseded by the HumanMethylationEPIC BeadChip micro-array (EPIC). The EPIC array has substantial overlap with the 450K and extends genome coverage to almost twice the number of loci (Moran *et al.*, 2016). With this increase in size of data it is apparent that current methodologies are not suitable for handling the large memory requirements necessary for analysis.

Analysis of DNA methylation array data is usually performed using one of three software packages: Minfi (Aryee *et al.*, 2014), ChAMP (Morris *et al.*, 2014) and RnBeads (Assenov *et al.*, 2014), all available on Bioconductor (Gentleman *et al.*, 2004). Minfi provides tools for the reading-in of raw data files, normalization, mapping of DNA methylation data to the genome and the identification of differentially methylation positions and regions. The ChAMP package extends the minfi package but also seeks to integrate other analyses and incorporates a selection of useful tools such as batch correction and gene enrichment analysis into a rigid workflow. RnBeads also offers a similar workflow to ChAMP but is not limited to DNA methylation micro-array data and can additionally analyze sequencing data. RnBeads also seeks to guide users through analyses with sequential functions and can even perform an entire analysis pipeline within a single function. Other packages worth mentioning include MethylAid (van Iterson *et al.*, 2014) and wateRmelon (Pidsley *et al.*, 2013), which focus on the quality control and preprocessing of DNA methylation data respectively. WateRmelon is extremely compatible with minfi, ChAMP and RnBeads and provides a variety of useful normalization methods and quality control tools. MethylAid thoroughly examines the control probes located on DNA methylation micro-arrays and presents users with a collection of graphics that help diagnose problematic samples. Downstream analysis of any resultant processed data is performed on a probe-by-probe basis with tools such as limma (Ritchie *et al.*, 2015) or with a variety of methods to identify differentially methylated regions such as bumphunter (Jaffe *et al.*, 2012) or block-finding (Hansen *et al.*, 2011).

Analysis of DNA methylation data from the raw format (.idat files) first requires the parsing of data using the illuminaio package (Smith *et al.*, 2013) and conversion into a useful format. Using the minfi package as an example: idat files are read into R, into memory, as an RGChannelSet object and subsequently can be converted into a MethylSet object using a specified normalization methodology or left unprocessed whilst simultaneously matching probes to identifiers. This MethylSet object essentially contains two matrices corresponding to methylated (*M*) and unmethylated (*U*) intensities. Statistical analysis of DNA methylation data mostly involves *β* values which are the ratio between the Methylated and total signals, defined as $\beta =\frac{M}{\left(M+U+\alpha \right)}$, where *α* is an arbitrary value to offset low intensity values (usually 100). Assuming all three steps (RGChannelSet → MethylSet → *β* matrix) are performed within a single R session it would not be unreasonable to assume that there are three copies of the same information stored in memory. If such analysis was performed on a dataset consisting of 1000 450K arrays we can expect to require at least 16 GB of memory (Supplementary Materials S1) to simply load and convert data from raw format to a biologically interpretable output before any statistical analysis has been performed. The memory requirements may be mitigated through careful memory management and garbage-collection however taking such steps would require reloading data into memory if they are needed at a later point in time.

All the R packages described require data to be first loaded into memory prior to any analysis. This can become an issue when handling particularly large datasets as this would take up a considerable amount of time and memory (depending on the computer) to load into R. Presently, this is not an issue as the average size of an experiment using 450K arrays is around 100 samples (400 Mb *β* matrix size). Out of the 900 experiments deposited onto GEO (as of July 2017), only 27 of these have sample sizes larger than 500 and these larger studies (Hannon *et al.*, 2016; Jaffe *et al.*, 2016; Liu *et al.*, 2013) may have been presented with analytical challenges during down-stream analysis. Furthermore, large-scale analyses that involved the aggregation of numerous datasets such as the ones used in creating the epigenetic clock (Horvath, 2013) or exploring repositories such as Marmal-Aid (Lowe and Rakyan, 2013) may have been severely limited by the need to load all the data into memory as this would have made analysis computationally expensive.

Recent efforts have been made to handle this problem, notably with the release of the meffil R package (Min *et al.*, 2017). The meffil R package allows the parsing of data one sample at a time and offers a single form of normalization (functional normalization) but is still limited by the fact that end result, *β* values, are stored in memory. In addition to this, meffil does not permit for the (raw) methylated and unmethylated intensities to available alongside the *β* values which can be useful in certain analyses. Furthermore meffil does not allow for interactive preprocessing of data prior to normalization, a feature that is highly important in our experience of EWAS studies.

This feature of analysis, coupled with the release of the EPIC array means that data will be increasing in size and current methodologies may not be suitable for the analysis of large datasets. To combat potential memory constraints imposed by DNA methylation analysis we introduce the bigmelon R package which includes memory-efficient tools for reading-in, quality control, exploring data and provides a practical workflow. In a well-run large-scale genomics project the data is examined, quality-controlled and stored as experimental batches are produced, rather than at the end. Bigmelon is the only existing package that is designed to facilitate a workflow of incremental data addition and analysis.

## 2 Approach

The bigmelon package makes use of the genomic data structure file format (.gds format) implemented in the gdsfmt package (Zheng *et al.*, 2012). Originally designed for the storage of SNP micro-arrays used in GWAS, the.gds format is a hard-disk representation of data with libraries that support efficient access. The gdsfmt package is also used by the GWASTools package (Gogarten *et al.*, 2012) and the SNPRelate package (Zheng *et al.*, 2012) which provide tools for principal components analysis and identity-by-descent algorithms that are integral in GWAS for adjusting for population structure and cryptic relatedness. In a similar manner, bigmelon is an extension of both gdsfmt and wateRmelon that enables the analysis of high dimensional DNA methylation data. The design objective of bigmelon is to provide the tools necessary for a complete workflow, these include quality control, normalization and statistical testing but also provide methods for further evaluation and analysis. Tools are additionally provided for estimating covariates such as age (Horvath, 2013), sex and whole blood cell-type proportions (Houseman *et al.*, 2012). Another heavily used tool for evaluating and exploring data is principal components analysis, and an efficient sampling approach to doing this on a large datasets is provided. Finally, the package is designed to facilitate incremental analysis, so that small batches of data can be readily looked at for quality control and even allow for first pass analyses as data is produced.

## 3 Materials and methods

**Fig. 1.**
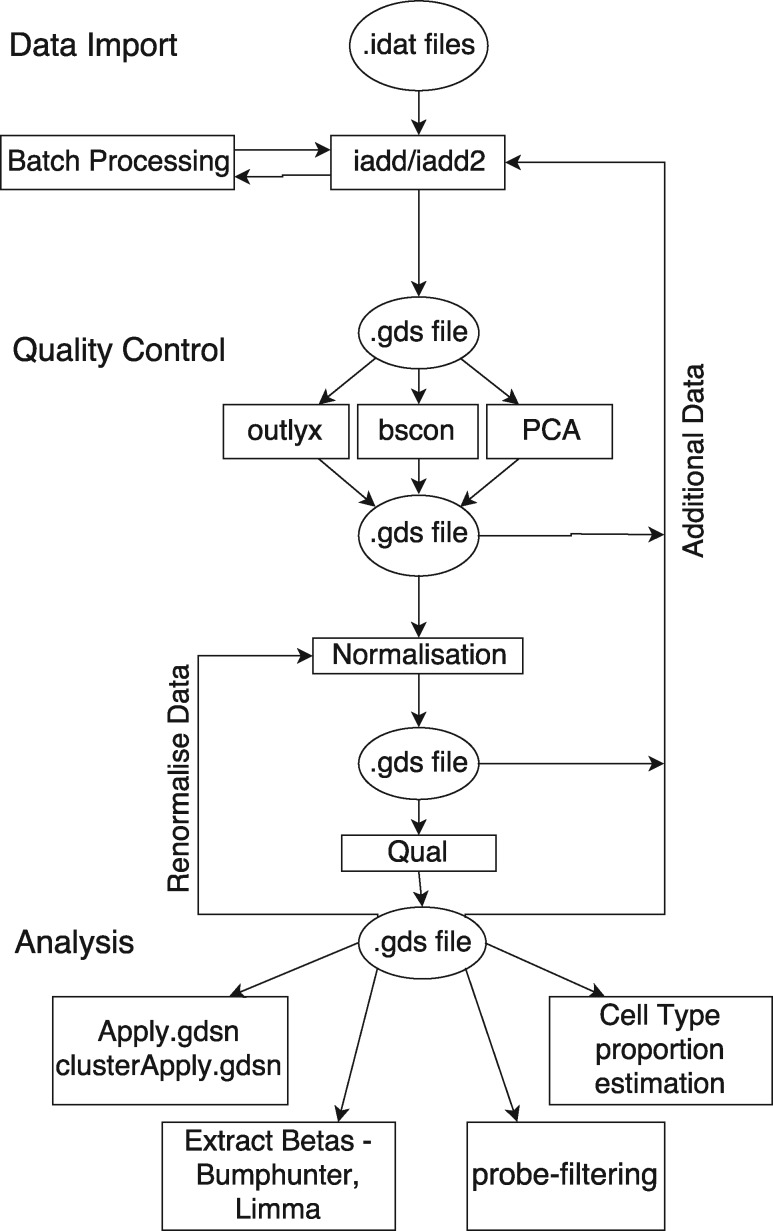
Example of bigmelon workflow. The workflow is broken up into three parts: Data Import, Quality Control and Analysis. Quality-control and analysis boxes propose examples that can be used at each stage of the analysis

A summary of the bigmelon workflow is described in Figure 1, the workflow can be broken down into three main parts: data import, quality control & preprocessing and analysis. Further descriptions of each section are as follows:

1. **Data import**: Much like other packages described, bigmelon offers the ability to read data into R into the gds file format using the iadd or iadd2 functions. The output of these functions is a hard-disk representation of an object that closely resembles the methylumiset object from the methylumi package (Triche *et al.*, 2013). For large data-sets these functions support memory-efficient batch processing. minfi (RGChannelSet, MethylSet) and methylumi (MethyLumiSet) objects can also be converted into gds format using the eset2gds function.
2. **QC & preprocessing**: Once data is in a gds file, it is possible to do thorough quality control using a number of memory-efficient tools. These include checking for outliers (outlyx), array quality (bscon), principal components analysis and age predictions, which can reveal mislabelling and other problems. After problematic samples are removed the data can be normalized. A range of quantile normalization methods are available as in wateRmelon. We introduce (qual), a quality measure based on the magnitude of changes introduced by the normalizer. This can identify further problematic samples which can degrade the quality of the dataset, for example introducing test-statistic inflation.
3. **Analysis**: One way to analyze the data is to extract the *β* values or subsets of them from the gdsfmt object and analyze them with any of the conventional methods. Bigmelon also facilitates conversion to MethylSet and MethyLumiSet objects using the gds2mset or gds2mlumi functions. This of course will be limited by the available memory. The core of EWAS analysis probewise analysis, and this is can be done relatively fast using minimal memory with apply.gdsn, and can also be parallelized using clusterApply.gdsn More complex analysis methods can be adapted for use with bigmelon objects. We provide a guide to doing this using bumphunter, and a bumphunter method is provided in the package.

### 3.1 Datasets

To demonstrate the capability of the bigmelon package we analyze two large datasets. The first consists of 1193 individuals from the *Understanding Society:* UK Household Longitudinal Survey. The goal of *Understanding Society* is to assess long-term and short-term effects of social and economic change on a variety of outcomes. Social and economic data are recorded through questionnaires and additional information including biomarker data and genotyping micro-arrays have also been obtained. Biomarker and relevant questionnaire data are available at https://www.understandingsociety.ac.uk/about/health/data upon request. 500Ng of whole blood DNA from each individual was treated with sodium bisulfite using the EZ96 DNA methylation kit (Zymo Research, CA, USA) following manufacturer’s standard protocol. DNA methylation intensities were assess using Illumina Infinium HumanMethylationEPIC BeadChips (Illumina Inc, CA, USA) in the Laboratory of Professor Jon Mill (University of Exeter). DNA methylation levels were assessed on an Illumina HiScan System (Illumina). This data-set is used to demonstrate the complete workflow described in Figure 1.

The second dataset is the Marmal-aid database (Lowe and Rakyan, 2013). Marmal-aid is the largest, most readily available dataset for DNA methylation consisting of 14 586 450K arrays. Originally it was collated to be used as a reference database for many cancerous and noncancerous tissues as it contains rich detail about each array (Tissue, Disease State, Sex and Age) but it can also serve as a useful resource for software performance on very large datasets.

### 3.2 Comparisons of memory usage

To test the difference in memory usage during analysis we the normalizeQuantiles function from limma (used on the Marmal-Aid dataset) with the bigmelon optimized versions (qn.gdsn). Bigmelon contains many optimized versions of functions used to normalize data and reproduce the results of the analysis precisely but differ in how the computations are handled. The aim of testing the difference in memory usage is to demonstrate that it is possible to execute memory expensive computations without much cost of speed. Memory usage was recording using an in-house bash script (Supplementary Materials S2) to monitor the memory usage of a specified R process at regular intervals during the normalization process.

### 3.3 Data accession

To estimate how much time it takes to retrieve that data from the hard disk into memory, the time taken to retrieve random portions of data from the Marmal-Aid dataset using the microbenchmark package (Mersmann, 2015).

All analyses were performed using R 3.4.0 on a machine with 500GB RAM (necessary for conventional analysis).

## 4 Results

### 4.1 Bigmelon provides a convenient workflow

Data import: the functions iadd and iadd2 conveniently read in raw data (idat files), and can append new data to an existing gdsfile, which is the key mechanism allowing an incremental workflow. We go through an analysis of Understanding Society data-set to demonstrate the steps shown in Figure 1.

**Fig. 2.**
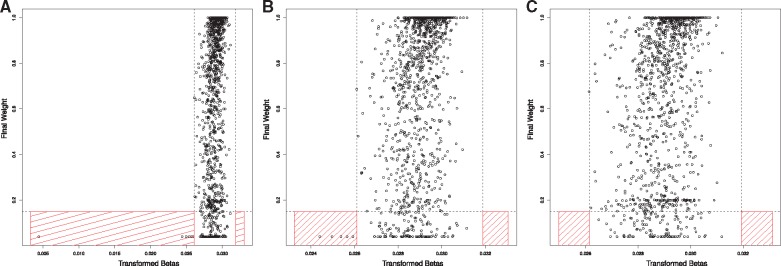
Demonstration of outlyx on Understanding Society Dataset (*n* = 1193). (**A**) The results of outlyx used on all samples, (**B**) The results of outlyx with an obvious outlier removed and (**C**) the results of outlyx with all outliers removed

Quality Control: The outlyx function is a robust outlier detection tool that identifies outlying samples without supervision (Fig. 2). Within the original 1193 samples it can be seen that 6 samples are outlying (Fig. 2A), and removal of the most-outlying sample yields no change in the results for the remaining samples (Fig. 2B), suggesting that the tool is not susceptible to swamping/masking effects. Similarly, removal of all outlying samples does not unmask further candidates. (Fig. 2C) further demonstrating the robustness of the quality-control procedure. Due to the unsupervised nature of this tool, it can also be used to check data-sets after quality control.

Atypical arrays are most likely the result of DNA quality or processing faults, and the control probes on the array offer some information on this. bscon calculates a bisulfite conversion value, which would ideally be 100%. In some datasets this may be lower, but certainly particularly low samples are an indication of trouble. Supplementary Figure S1 shows the output of bscon on the Understanding Society dataset. Here we select a conservative threshold of 85% bisulfite conversion, and six samples were identified as having low-quality, from these only one of these was also identified as being atypical using outlyx.

Minor systematic differences between arrays introduced by sample quantity or other technical variations are readily normalized away, and quantile normalization based methods are excellent for imposing identical distributions on vectors that are similar in the first place. The objective of EWAS is to detect a relatively small number of true differences on a homogeneous background. We introduce a function qual that measures ’normalisation violence’ required to bring an individual array into line. The properties of the measure have not been fully explored, but a reasonable cutoff of 0.05 for root mean squared deviation identifies 6 potentially bad arrays in this dataset (Supplementary Fig. S2).

In summary, out of 1193 samples we began with, 18 were removed for failing qc criteria, 6 from outlyx, 7 from qual and 5 from bscon as detailed above. Each of these involve thresholds that may need to be that may need to be adjusted in some cases but in the main they can be used as automated filters. Additional qc and sanity checks are equally important but require more human intervention. Principal component analysis often reveals stratification, samples with the wrong labelled sex and other problems. In Supplementary Figure S3 we present the first and second principal component loading values which clearly show two clusters which can be used to guess the sex of samples, in our experience we have found that the number of probes required to produce such a plot is small and in some cases <1% of the total number of probes on a micro-array will produce a biologically interpretable result. It is for that reason the principal components method packaged in bigmelon allows for a random selection of probes to be used instead of the full data-set. Age prediction (Supplementary Fig. S4) can also be used to check whether or not samples aligned with their supposed phenotypic data. In addition to offering age prediction using Horvath’s coefficients we also allow the option to compute ages using Hannum’s coefficients (not shown) (Hannum *et al.*, 2013).

Cell-Type composition estimation (Supplementary Fig. S5) has been optimized by imposing methylated and unmethylated quantiles onto the reference dataset instead of normalizing the reference and biological dataset together as it was felt that given a large enough number of samples, the addition of the reference dataset would not have an effect on the precision of the cell-type estimates. When compared to minfi it can be seen that the cell counts calculated using by normalizing data together do not vary much from the cell counts calculated from the alternative method and correlated highly together (Root Mean Squared Differences between minfi and bigmelon estimated cell counts range from 0.020 to 0.006).

### 4.2 Bigmelon uses less memory

**Fig. 3.**
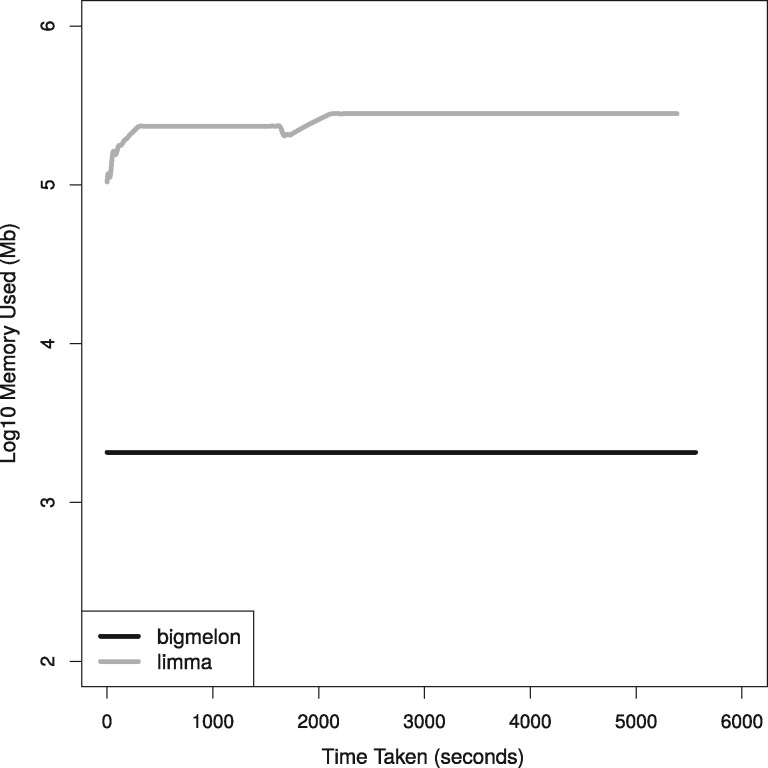
Comparison of quantile normalization on 52 GB *β* matrix from Marmal-aid dataset (*n* = 14 586) using limma::normalizeQuantiles function and bigmelon::qn.gdsn, computation was performed on a single core computer with 500GB of memory

When comparing the memory usage of bigmelon to other software (limma and wateRmelon) it can be seen that there is at least a hundred-fold difference in memory usage at any given time throughout analysis (Fig. 3). These improvements in memory efficiency are mostly dependent on the size of the data that are being analyzed however this demonstrates that there is vast improvement using two large biological data-sets. This improvement suggests it may be possible to carry out a complete analysis workflow on a low-end computer (e.g. a workstation with just over 2 GB memory) as a full analysis only requires 600 MB of memory at any given time. In this comparison the performance of limma is identical to that of wateRmelon and minfi, as all use the same normalizeQuantiles function. This is further demonstrated in Supplementary Figure S6 where we assess the time it takes to quantile normalize varying data-sizes on a modest workstation where it quickly runs out of memory and resorts to thrashing to complete analysis. This reflects how both minfi, wateRmelon and other R packages would perform.

### 4.3 Random access is fast

**Fig. 4.**
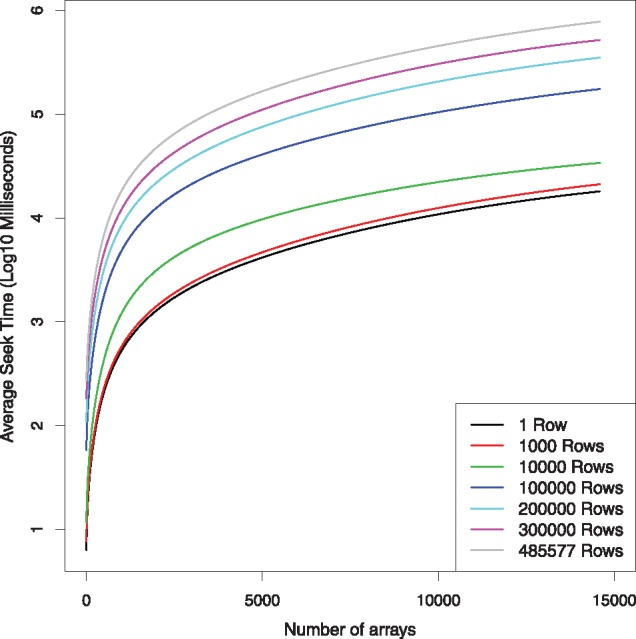
Median time spent randomly accessing different sized portions of data from the Marmal-Aid data-set (*n* = 14 586) stored in.gds file format

Despite being stored on the hard-disk access is still relatively fast (Fig. 4). The median seek-time, using the Marmal-Aid dataset as a benchmark, ranged from 6.2 ms when seeking a single data-point randomly from the gds file to 13 min, when seeking all the data (458 877 rows, 14 586 columns). Additionally, accessing full rows and columns from hard-disk take on average 14 and 0.3 s respectively. It however must be noted that it appears the time required for accessing any amount of data is dependent on the number of samples being accessed at the time, for example accessing all data for a 500 sample dataset will only take 22 s.

## 5 Discussion

We have demonstrated how the bigmelon package resolves a severe limitation that is associated with current methodologies in EWAS. The bigmelon package facilitates the reading-in, quality-control, preprocessing and statistical analysis of DNA methylation micro-array data with an additional selection of useful tools. Through storage of data on the hard-disk it is possible to circumvent majority of memory constraints and allow the analysis to be performed on most computers. Additionally, due to the nature of the workflow (Fig. 1) it is possible to append data to pre-existing gds files allowing users to analyze data as it is produced. The workflow has similarities to the workflows presented in minfi and ChAMP, and there is a reasonably simple transition path from these to bigmelon.

Currently, bigmelon does not support all of the generalized clustering methodologies used for the identification of differentially methylated regions, although we do have an implementation of bumphunter. Bigmelon allows for the seamless transition to and from minfi or methylumi data structures (MethylSet and MethyLumiSet objects), offering a route to using specialized tools if enough memory is available. To assist in the writing optimized functions for users with highly specific analyses we have provided a guide to writing functions for bigmelon that covers most of the important aspects to writing memory efficient code (Supplementary Materials S3). We plan to implement as many analyses as we see fit and will strive towards implementing many existing methodologies in the future.

## 6 Conclusion

The bigmelon package offers users the ability to easily handle and analyze large DNA methylation datasets (both 450K and EPIC) without the need of huge RAM or powerful computers however can reap the benefits of powerful computers as the gds file format supports parallel computing. The bigmelon package trivializes the compilation, exploration and analysis of extremely large datasets and should prove integral for the analysis of DNA methylation data in the future.

## Supplementary Material

Supplementary Data
