# Supplementary material for: Bigmelon: tools for analysing large DNA methylation datasets
Source: Bioinformatics. 2018 Aug 23;35(6):981–6. doi: 10.1093/bioinformatics/bty713 (PMC6419913; doi:10.1093/bioinformatics/bty713)
Supplement: Supplementary Data [file bty713_supp.zip › bty713-suppl_data/Suppl_Materials_3_Writing_Bigmelon_Functions_Guide.pdf]

Writing bigmelon-ised Functions

Tyler J. Gorrie-Stone

Preface

This is an introduction to implementing functions for bigmelon. Given enough memory, it's straightforward to extract the complete intensity or beta metrix from a .gds file and work on that. In order to make a function that works memory-efficiently on large datasets though, you need to think about how the function works, and what subsets you should extract to work on sequentially or in parallel.

We will demonstrate using `bumphunter::bumphunter` as an example and the six 450k samples from the minfiData package as example data.

We will assume you have also read the package vignettes.

```
library(bigmelon)
library(parallel)       # optional, for parallel processing examples
library(microbenchmark) # optional, to demonstrate some code performance
library(bumphunter)
library(minfiData)
  # make a gdsfile
  bd <- system.file('extdata', package='minfiData')
  gfile <- iadd2(bd, gds = 'melon.gds')
  closefn.gds(gfile)
  # open the file again, allowing forking (important for multicore processing)
  gfile <- openfn.gds('melon.gds', allow.fork = T)
```

Accessing Data, loops and `apply`

Preprocessing steps such as quantile normalisation, tend to involve processing an array at a time, ie looping over columns or `apply` on margin=2. Analyses are more often probewise, ie looping over rows or `apply` on margin=1. In either case it is important to analyse what the function has to keep from these operations and whether that has to be kept in a memory-efficient form. This generally comes down to whether it is a column or row summary (ie output is one or a few rows/columns) or the same shape as the input. There is an `apply.gdsn` function that optionally keeps the output as a gdsfile node.

Because of the overhead of file access, it's also worth considering combining several operations into a pass over the matrix instead of making several pass

Accessing Data

Within `bigmelon` we provide user friendly `[` functions to enable users to directly access data similar to that of an `expressionSet` object like a `MethylSet` object. This is described, in the vignette. These are particularly useful for interactive use if you are inclined to look at certain regions.

```
# Pulling out the first row of the data-set
betas(gfile)[1, 1:4, name = TRUE]
```

```
## 5723646052/5723646052_R02C02 5723646052/5723646052_R04C01
##                    0.4143280                    0.3733613
## 5723646052/5723646052_R05C02 5723646053/5723646053_R04C02
##                    0.2125911                    0.1893959
```

```r
# Alternative
gfile[1, 1:4, node = 'betas', name = FALSE]
```

```
## [1] 0.4143280 0.3733613 0.2125911 0.1893959
```

There is little difference between the two above examples. You can access all data as you normally would using logical, character or numerical indexing. A key distinction is the `name` argument will provide the dimnames of the resultant vector/matrix. In the first example we are using a familiar function `betas` on `gfile` and then indexing. While in the second example we are calling the `gfile` object directly and adding an additional argument within the `[` function to call a specific node that we are interested in selecting. This is particularly useful for calling data that does not have a standard name or a function associated with it.

Alternatively you can use `readex.gdsn`, which `[` calls, directly. In most cases this is faster but requires a list of indices and does not provide dimnames

```r
node <- index.gdsn(gfile, 'betas') # target specific node of interest
readex.gdsn(node = node, sel = list(1, 1:4))
```

```
## [1] 0.4143280 0.3733613 0.2125911 0.1893959
```

Lastly, and most importantly, accessing data by column is considerably faster than accessing data by row! So in terms of performing analysis if you can restructure the code to handle columns instead of rows the time spent accessing data is greatly reduced.

Looping Examples

Now that we know how to access data we can begin with some loops. Here we will compare a few ways that a for-loop can be done - and evaluate the caveats of each, and then compare it with the `apply`-like functions in `gdsfmt`. In these examples we will emulate `colSums` for a gds object.

```r
# Example 1 using `[`
sums1 <- function(gfile){
  sums <- vector('numeric', length(colnames(gfile)))
  for(i in seq_along(colnames(gfile))){
    sums[i] <-sum(betas(gfile)[,i], na.rm = TRUE)
  }
  sums
}
microbenchmark(sums1(gfile), times = 10)
```

```
## Unit: seconds
##          expr      min       lq     mean   median       uq     max neval
##  sums1(gfile) 1.428896 1.437342 1.473207 1.484138 1.501822 1.52143    10
```

```r
# Example 2 using readex.gdsn
sums2 <- function(gfile){
  sums <- vector('numeric', length(colnames(gfile)))
  for(i in seq_along(colnames(gfile))){
    sums[i] <- sum(readex.gdsn(index.gdsn(gfile, 'betas'), sel = list(NULL, i)), na.rm = TRUE)
  }
  sums
}

microbenchmark(sums2(gfile), times = 10)
```

```
## Unit: milliseconds
##          expr     min       lq   mean   median       uq      max neval
```

```
##  sums2(gfile) 29.4314 29.51255 30.396 29.56546 29.92343 36.90798    10
```

The time difference between `[` and using `readex.gdsn` is noticeable, while it is relatively small here it could be a problem in larger data-sets.

Alternatively the same result can be achieved with `sapply`

```
sums3 <- function(gfile){
  sums <- sapply(seq_along(colnames(gfile)), function(i, gfile){
    sum(readex.gdsn(index.gdsn(gfile, 'betas'), sel = list(NULL, i)), na.rm = TRUE)
  }, gfile = gfile)
  sums
}
microbenchmark(sums3, times = 10)
```

```
## Unit: nanoseconds
##   expr min lq  mean median uq  max neval
##  sums3   1 70 329.1     71 71 2724    10
```

apply-like functions

The `apply.gdsn` is **usually** faster than any for-loop in R and has added benefits that it can store the output directly into a gds node should you prefer it, usually this allows for cleaner code and looks nicer in my opinion. I highly recommend reading the manual pages for `apply.gdsn`! Here we are also able to compute the `colSums` of the small matrix in rapid time.

```
sums4 <- function(gfile){
  sums <- apply.gdsn(node = index.gdsn(gfile, 'betas'),
    margin = 2, # colSums
    FUN = sum,
    selection = NULL,
    # Otherwise selection can be a list akin to readex.gdsn
    as.is = "double",
    # Can be "list", "none", "character", "logical", "gdsnode"
    na.rm = TRUE # Other arg for sum!
  )
  sums
}

microbenchmark(sums4, times = 10)
```

```
## Unit: nanoseconds
##   expr min lq   mean median uq  max neval
##  sums4  70 70 1055.4     71 71 9849    10
```

This is comparible with the other examples, however the benefit of using `apply.gdsn` lies in its ability to process data by rows. (see below).

```
sums5 <- function(gfile){
  sums <- apply.gdsn(node = index.gdsn(gfile, 'betas'),
    margin = 1, # rowSums
    FUN = sum,
    selection = NULL,
    # Otherwise selection can be a list akin to readex.gdsn
    as.is = "double",
    # Can be "list", "none", "character", "logical", "gdsnode"
    na.rm = TRUE # Other arg for sum!
```

```r
  )
  sums
}

microbenchmark(sums5, times = 10)
```

```
## Unit: nanoseconds
##   expr min lq  mean median uq  max neval
##  sums5  70 70 217.4     71 71 1468    10
```

As we can see, `apply.gdsn` wastes little time in computing the `rowSums` of a matrix. If we were to do this with a for loop, we would be here for a very long time.

Depending on what you are using `apply.gdsn` for it is usually possible to parallelise it by replacing it with `clusterApply.gdsn` or writing a `mclapply` function. **n.b.** `clusterApply.gdsn` has problems when being used within functions (and in this Rmarkdown document and cannot be demonstrated but below is an example of how to use it). Also doing things in parallel will use more memory.

Another distinction of `clusterApply.gdsn` is that is cannot write data to a gds file, if you ever wish to do this; you will need to use a for loop or `apply.gdsn`.

```r
  cl <- makeCluster(2)
  sums <- clusterApply.gdsn(cl = cl,
     gds.fn = gfile[[1]],
     # gfile[[1]] is the absolute path of the gdsfile
     node.name = "betas",
     margin = 1,
     FUN = sum,
     selection = NULL,
     as.is = 'double'
   )
stopCluster(cl)
```

```r
mcsums <- function(gfile){
  sums <- mclapply(seq_along(colnames(gfile)), FUN = function(i, gfile){
     sum(readex.gdsn(
        index.gdsn(gfile, 'betas'),
        sel = list(NULL, i)),
        na.rm = TRUE
     )
   }, gfile = gfile, mc.cores = 2)
   sums
}
microbenchmark(mcsums(gfile), times = 10)
```

```
## Unit: milliseconds
##           expr      min       lq    mean   median       uq      max neval
##  mcsums(gfile) 84.38329 113.0161 120.896 127.6162 135.2787 142.0332    10
```

There's a balance between the added hassle of parallelising methods and the speedup that it produces. For most analyses to date we have managed without it.

A Note

Some operations may not be parallelisable. These include copying data from one gds-object to another, doing operations that require the results of a previous iteration and writing data to a gds file. In general

terms, always try to do things using `apply.gdsn` or `clusterApply.gdsn`. If you cannot move onto a for-loop, sapply, mclapply etc., if you are attempting to iterate over rows consider chunking the matrix into 1000 by p matrix and load and process each chunk into RAM instead of loading the individual row (loading a small chunk (up to 1000 rows) is just as fast loading a single row). If all else fails, bite the bullet and load the entire matrix into memory or find a heuristic approach to the problem.

An example of optimisation: bumphunter

We continue to implement bigmelon methods for popular EWAS realated functions, but can't anticipate all users' needs. It is often possible to do this yourself without rewriting very much of the code.

We will be optimising the `bumphunter` function. The code is quite long and there is a lot to go through but I will try to describe some of my thought process behind the optimisation.

Review the Code:

The bumphunter function is an interesting function and heavily used in the realms of EWAS. What distinguishes it from other functions is that it computes its own test statistics and provides two cross-validation methods for the tools. As a result it involves a lot of matrix arithmetic on the entire dataset. I of course am not the original author of the code and the coments and documentation can be a huge help in breaking the problem down.

After some careful review I was able to narrow down that the optimisation of bumphunter could be acheived by rewriting two parts of the function. Specifically these parts required the entire dataset. With the current test data it is a 485577 x 6 matrix) but in the intended use case it could be multiple Gb.

To begin optimisation we can start by making a direct copy of the code and strip out any of the preliminary checks that consider data sanity (e.g `stopifnot(is.matrix(data))`).

I made the following changes to the start of the code:

```
n <- objdesp.gdsn(mat)$dim[1] # new
p <- objdesp.gdsn(mat)$dim[2] # new
#if (!is.matrix(mat))
#  stop("'mat' must be a matrix.")
if (p != nrow(design))
  stop("Number of columns of 'mat' must  match number of rows of 'design'")
```

Maybe I should have used better names than n and p, but it's useful to have the dimensions stored at the beginning and to check they make sense.

Step 1: 'Permutation'

Moving onto the first piece of code we will need to optimise: This is the `.getEstimate` function - which as the name implies computes the beta estimates for the model you intend to run. The code is very fast and it is a shame that we need to break it down into something slower to make it memory efficient.

Here is what I came up with (Changes I have made I have indicated with a `##` the line above:

```
.getEstimate2 <- function(mat, design, coef, B = NULL, permutations = NULL, full = FALSE){
  ##
  p <- objdesp.gdsn(mat)$dim[2]
  ##
  n <- objdesp.gdsn(mat)$dim[1]
  v <- design[, coef]
```

```
A <- design[, -coef, drop = FALSE]
qa <- qr(A)
S <- diag(nrow(A)) - tcrossprod(qr.Q(qa)) # ncol * ncol matrix, "small"
vv <- if(is.null(B)){
  matrix(v, ncol = 1)
} else {
  if (is.null(permutations)) {
    replicate(B, sample(v))
  } else {
    apply(permutations, 2, function(i) v[i])
  }
}
sv <- S %*% vv
vsv <- diag(crossprod(vv, sv))
#b <- (mat %*% crossprod(S, vv))/vsv
#  if (!is.matrix(b))
#      b <- matrix(b, ncol = 1)
if(full){
  # sy <- mat %*% S
  df.residual <- p - qa$rank - 1
  if(is.null(B)){
  ## New Chunk
    o <- apply.gdsn(node = mat, margin = 1, as.is = 'list',
      FUN = function(x, S, vv, vsv, sv, df.residual){
        sy <- x %*% S
        b <- (x %*% crossprod(S, vv))/vsv
        tcross <- tcrossprod(b, sv)
        sigma <- sum((sy - tcross)^2)/df.residual
        list('B'=b, 'sigma'= sigma)
      }, S = S, vv = vv, vsv = vsv, sv = sv, df.residual = df.residual
    )
  } else {
    o <- apply.gdsn(node=mat, margin=1, as.is = 'list',
      FUN = function(x, S, vv, vsv, sv, B, df.residual){
        tmp <- sy <- x %*% S
        sigma <- b <- (x %*% crossprod(S, vv))/vsv
        for(j in seq_len(B)){
          tmp <- tcrossprod(b[,j], sv[,j])
          sigma[j] <- sum((sy-tmp)^2)
        }
        sigma <- sqrt(sigma/df.residual)
        list('B'= b, 'sigma'=sigma)
      }, S = S, vv = vv, vsv = vsv, sv = sv, df.residual = df.residual, B = B
    )
  }
  coef  <- if(is.null(B)) sapply(o, '[[', 'B') else t(sapply(o, '[[', 'B'))
  sigma <- if(is.null(B)) sapply(o, '[[', 'sigma') else t(sapply(o,
     '[[', 'sigma'))
  out <- list(coef = coef, # n * B big
      sigma = sigma, # n * B big
      stdev.unscaled = sqrt(1/vsv),
      df.residual = df.residual)
  if(is.null(B)) out$stdev <- as.numeric(out$stdev)
```

```
  } else {
    out <- apply.gdsn(node=mat, margin = 1, as.is = 'list',
       FUN = function(x, S, vv, vsv){
         b <- (x %*% crossprod(S, vv))/vsv
       }, S = S, vv = vv, vsv = vsv
    )
    out <- do.call(rbind, out)
  }
  ## End new Chunk
  return(out)
}
```

There is a lot to take in but we are certain the new function works!

```
mat <- betas(gfile)
design <- model.matrix(~c(1,1,1,2,2,2))
head(bumphunter:::.getEstimate(mat = mat[,] , design = design, coef = 2, B=NULL, full = F))
```

```
##                       [,1]
## cg00000029 -0.089415463
## cg00000108 -0.014796262
## cg00000109 -0.008447514
## cg00000165  0.182835398
## cg00000236  0.007545778
## cg00000289 -0.048587910
```

```
head(.getEstimate2(mat = mat, design = design, coef = 2, B=NULL, full = F))
```

```
##               [,1]
## [1,] -0.089415463
## [2,] -0.014796262
## [3,] -0.008447514
## [4,]  0.182835398
## [5,]  0.007545778
## [6,] -0.048587910
```

We must remember to at some point relabel the dimnames, this can be usually be done at the end.

There is a lot to unpack here. So we will begin at the top and work down:

The code is remarkably different from the original code (the parts that have been commented out). Most notably I have moved the two large cross products (`b <- (mat %*% crossprod(S, vv))/vsv` and `sy <- mat %*% S`) within `apply.gdsn`, and modified the structures of the code so that they will compute the crossproduct of a single row.

Taking a closer look at one of the `apply.gdsn`'s being used here…

```
... # Rest of code above
      o <- apply.gdsn(node=mat, margin=1, as.is = 'list',
          FUN = function(x, S, vv, vsv, sv, B, df.residual){
            tmp <- sy <- x %*% S
            sigma <- b <- (x %*% crossprod(S, vv))/vsv
            for(j in seq_len(B)){
              tmp <- tcrossprod(b[,j], sv[,j])
              sigma[j] <- sum((sy-tmp)^2)
            }
            sigma <- sqrt(sigma/df.residual)
            list('B'= b, 'sigma'=sigma)
```

```
          },
          S = S, vv = vv, vsv = vsv, sv = sv,
          df.residual = df.residual, B = B
      )
    coef <- if(is.null(B)) sapply(o, '[[', 'B') else t(sapply(o, '[[', 'B'))
    sigma <- if(is.null(B)) sapply(o, '[[', 'sigma') else t(sapply(o, '[[', 'sigma'))
    out <- list(coef = coef, # n * B big
        sigma = sigma, # n * B big
        stdev.unscaled = sqrt(1/vsv),
        df.residual = df.residual)
... # Rest of code
```

The structure is somewhat similar to a regular `apply` or `lapply` but with a few differences. The `as.is` specifies the output format, this can be numeric, character, a list or a gdsnode (which we will see later).

Here we can see that for each row of `mat` we compute using the `%*%` and then convert the output into the correct format at the end of the apply. Since the output of `.getEstimate` is at minimum 2 matricies of length n, and B columns. This is fairly small in terms of memory usage, so we are comfortable with keeping this in memory. If we suspect that we would have a `B > 1000` then we may want to consider storing the output into a gds file, and thus we would need to change the code to store a large matrix. We provide the `apply.gdsn` with static elements of the function such as `S`, `vv`, etc. so that we do not have to continuously recalculate them as this can eat into computation time, expecially when these variables are quite small.

Step 2: Bootstrapping

After computing the estimates, we need to do the null boot-strapping or null permutations. We already optimised the permutation step by updating `.getEstimate` so we can look towards the boot-strapping part of bumphunter. The bootstrapping section makes use of the `foreach` package to do some multicore processing if specified to, but we will initially do the analysis on a single core.

Here is what I came up with:

```
if (nullMethod == "bootstrap"){
  message("[bumphunterEngine] Performing ", B, " bootstraps.")
  qr.X <- qr(design)
  ##rescale residuals
  h <- diag(tcrossprod(qr.Q( qr(design))))
  ##create the null model to which we add bootstrap resids
  design0 <- design[,-coef,drop=FALSE]
  qr.X0 <- qr(design0)
  ##
  boots <- createfn.gds('bs.gds', allow.duplicate = TRUE)
  res <- add.gdsn(node = boots, name='resids', val = NULL, storage = 'float64',
                  valdim = c(p,0))
  null <- add.gdsn(node = boots, name='null', val = NULL, storage = 'float64',
                  valdim = c(p,0))
  apply.gdsn(node = mat, margin = 1, as.is = 'gdsnode', target.node = list(x=res, y=null),
             FUN = function(x, s1, s2, n1){
               res <- t(s1 %*% x)/s2
               null <- t(n1 %*% x)
               list(x=res, y=null)
              }, s1 = t(diag(nrow(design)) - tcrossprod(qr.Q(qr.X))),
                 s2 = sqrt(1-h), n1 = tcrossprod(qr.Q(qr.X0))
            )
```

```
  ##Now do the boostraps
  chunksize <- ceiling(B/workers)
  bootIndexes<-replicate(B, sample(1:p,replace=TRUE),simplify=TRUE)
#    tmp <- foreach(bootstraps = iter(bootIndexes, by = "column", chunksize = chunksize),
#     .combine = "cbind", .packages = "bumphunter") %dorng% {
#         apply(bootstraps, 2, function(bootIndex){
#             ##create a null model
#             matstar <- null+resids[,bootindex]
##              ##compute the null beta estimate
#            nullbetas <- backsolve(qr.R(qr.X),crossprod(qr.Q(qr.X),t(matstar)))[coef,]
#           if (useWeights){
#                 ##compute sigma
#                  sigma <- rowSums(t(tcrossprod( diag(nrow(design)) -
#                           tcrossprod(qr.Q(qr.X)),  matstar))^2)
#                   sigma <-
#                        sqrt(sigma/(nrow(design)-qr.X$rank))
#                     outList <- list(coef=nullbetas,sigma=sigma)
#                  } else {
#                       outList <- nullbetas
#                    }
#                     return(outList)
#                  })
#                           }

## replace the foreach...
  tmp <- lapply(seq_len(ncol(bootIndexes)),
    FUN = function(x, resids, null, s1,s2,s3,s4,s5,coef, useWeights){
      outList <- apply.gdsn(list(x=resids, y=null), margin=c(2,2), as.is='list',
        FUN = function(X, j, s1, s2, s3, s4, s5, useWeights, coef){
          # create null model
          matstar <- X$y + X$x[j]
          # compute estimate
          nullbetas <- backsolve(s1, crossprod(s2, matstar))[coef]
          if(useWeights) {
            # compute sigma
            sigma <- sqrt(sum((s4%*%matstar)^2)/s5)
            outList <- list(coef = nullbetas, sigma = sigma)
          } else {
            outList <- nullbetas
          }
          return(outList)
        }, j = bootIndexes[,x],
           s1 = s1,
           s2 = s2,
           s3 = s3,
           s4 = s4,
           s5 = s5,
           useWeights = useWeights,
           coef = coef)
         if(useWeights) return(list(coef = sapply(outList, '[[', 'coef'),
                                    sigma = sapply(outList, '[[', 'sigma')))
         else return(unlist(outList))
       }, resids = index.gdsn(boots, 'resids'),
```

```
          null = index.gdsn(boots, 'null'),
          s1 = qr.R(qr.X),
          s2 = qr.Q(qr.X),
          useWeights = useWeights,
          coef = coef,
          s3 = tcrossprod(qr.Q(qr.X)),
          s4 = t(diag(nrow(design))-tcrossprod(qr.Q(qr.X))),
          s5 =  (nrow(design) - qr.X$rank)
  )
## Done
if (useWeights && smooth) { # Here...
  bootRawBeta <- do.call(Map, c(cbind, tmp))$coef # or sapply(tmp, '[[' ,'coef')
  weights <- do.call(Map, c(cbind, tmp))$sigma
  } else {
  ##
  bootRawBeta <- sapply(tmp, '[[' ,'coef')
  weights <- NULL
  }
  NullBeta<-bootRawBeta
  rm(tmp)
  rm(bootRawBeta)
  ##
  closefn.gds(boots)
  unlink(boots[[1]])
}
```

In summary: I replace the `foreach` with an lapply and optimised the bootstraps with a funky `apply.gdsn`. So there is alot to go though.

Once more I will go through some interesting features:

```
      boots <- createfn.gds('bs.gds', allow.duplicate = TRUE)
      res <- add.gdsn(node = boots, name='resids', val = NULL,
         storage = 'float64',
         valdim = c(p,0)
      )
      null <- add.gdsn(node = boots, name='null', val = NULL,
         storage = 'float64',
         valdim = c(p,0)
      )
      apply.gdsn(node = mat, margin = 1, as.is = 'gdsnode',
         target.node = list(x=res, y=null),
         FUN = function(x, s1, s2, n1){
             res <- t(s1 %*% x)/s2
             null <- t(n1 %*% x)
             list(x=res, y=null)
         },
         s1 = t(diag(nrow(design)) - tcrossprod(qr.Q(qr.X))),
         s2 = sqrt(1-h), n1 = tcrossprod(qr.Q(qr.X0))
      )
```

In this chunk we create a new gds file to store some values in, since the output of these `%*%` is going to generate a matrix the same shape as our input. We use `apply.gdsn` with `as.is = 'gdsnode'` and add `target.node = list(x=res, y=null)`.

What is handy is we can label where each of the data goes in the list output to avoid confusion. Inside the

`apply.gdsn` we compute both the scaled residuals and the null model estimate in a row-wise manner (this is done in two memory intensive steps in bumphunter: `resids <- t(tcrossprod( diag(nrow(design)) - tcrossprod(qr.Q(qr.X)), mat))` and `null <- t(tcrossprod(tcrossprod(qr.Q(qr.X0)), mat))`) but in bigmelon we take it nice and slow. Again like above we provide non-trivial computations (again do not take up much memory) as arguments to the `apply.gdsn` to avoid having to compute the same thing hundreds of thousands of times.

The next chunk is where things get interesting…

```
tmp <- lapply(seq_len(ncol(bootIndexes)),
  FUN = function(x, resids, null, s1,s2,s3,s4,s5,coef, useWeights)
    outList <- apply.gdsn(list(x=resids, y=null), margin=c(2,2), as.is='list',
      FUN = function(X, j, s1, s2, s3, s4, s5, useWeights, coef){
        # create null model
        matstar <- X$y + X$x[j]
        # compute estimate
        nullbetas <- backsolve(s1, crossprod(s2, matstar))[coef]
        if(useWeights) {
          # compute sigma
          sigma <- sqrt(sum((s4%*%matstar)^2)/s5)
          outList <- list(coef = nullbetas, sigma = sigma)
        } else {
          outList <- nullbetas
        }
        return(outList)
      }, j = bootIndexes[,x],
         s1 = s1,
         s2 = s2,
         s3 = s3,
         s4 = s4,
         s5 = s5,
         useWeights = useWeights,
         coef = coef)
       if(useWeights) return(list(coef = sapply(outList, '[[', 'coef'),
                                  sigma = sapply(outList, '[[', 'sigma')))
       else return(unlist(outList))
    }, resids = index.gdsn(boots, 'resids'),
       null = index.gdsn(boots, 'null'),
       s1 = qr.R(qr.X),
       s2 = qr.Q(qr.X),
       useWeights = useWeights,
       coef = coef,
       s3 = tcrossprod(qr.Q(qr.X)),
       s4 = t(diag(nrow(design))-tcrossprod(qr.Q(qr.X))),
       s5 =  (nrow(design) - qr.X$rank)
)
```

We remove the foreach in the original function and replace it with an `lapply` to iterate of the bootstraps. Then within each bootstrap we call `apply.gdsn` and compute the null model and get the beta estimates. Similar to `.getEstimate2` we supply non-trivial computations to the functions as stored variables to avoid computing them many times.

I would like to draw interest to this line: `apply.gdsn(list(x=resids, y=null), margin=c(2,2),...` as it demonstrates one of the most impressive functionalities of `apply.gdsn`, similar to `mapply` where you give multiple variables to be looped over in a matrix we can likely specify more than one object to the first argument in `apply.gdsn` in this case we supply two `gdsn.class` nodes within a list under the names `x` and

y. This translates to the ability to call either of the two objects within the applied function by calling `X$x` and `X$y` respectively. This makes it impressively easy to write memory efficient functions that require more than one large matrix and is one of the biggest advantages to using `apply.gdsn` over other ways of looping.

The rest of the code remains unchanged from the original function.

Testing the finished product

In most scenarios you will be able to write some code that is able to reproduce the results you want without using a large amount of memory - this would be in particularly useful if you intend on doing analysis that is not possible on the large scale. The trade of is a considerable amount of speed though.

```
set.seed(1)
pos <- sample(1:100000, 485577, rep=T)
set.seed(2)
chr <- sample(as.character(1:22), 485577, rep = T)

out <- bumphunterEngine(betas(gfile)[,], design=model.matrix(~c(1,1,1,2,2,2)),
                        chr=chr, pos=pos, nullMethod = 'permutation',
                        B = 3, coef = 2, verbose = T, pickCutoff = T)
```

```
## [bumphunterEngine] Using a single core (backend: doSEQ, version: 1.4.4).

## [bumphunterEngine] Computing coefficients.

## [bumphunterEngine] Performing 3 permutations.

## [bumphunterEngine] Computing marginal permutation p-values.

## [bumphunterEngine] cutoff: 0.226

## [bumphunterEngine] Finding regions.

## Warning in regionFinder(x = beta, chr = chr, pos = pos, cluster =
## cluster, : NAs found and removed. ind changed.

## [bumphunterEngine] Found 10849 bumps.

## [bumphunterEngine] Computing regions for each permutation.

## Loading required package: rngtools

## Loading required package: pkgmaker

## Loading required package: registry

##
## Attaching package: 'pkgmaker'

## The following object is masked from 'package:S4Vectors':
##
##     new2

## The following object is masked from 'package:base':
##
##     isNamespaceLoaded

## Warning in FUN(newX[, i], ...): NAs found and removed. ind changed.

## Warning in FUN(newX[, i], ...): NAs found and removed. ind changed.

## Warning in FUN(newX[, i], ...): NAs found and removed. ind changed.
```

```
## [bumphunterEngine] Estimating p-values and FWER.
```

```r
out <- bumphunterEngine.gdsn(betas(gfile), design=model.matrix(~c(1,1,1,2,2,2)),
                             chr=chr, pos=pos, nullMethod = 'permutation',
                             B = 3, coef = 2, verbose = T, pickCutoff = T)
```

```
## [bumphunterEngine] Using a single core (backend: doSEQ, version: 1.4.4).
## [bumphunterEngine] Computing coefficients.
## [bumphunterEngine] Performing 3 permutations.
## [bumphunterEngine] Computing marginal permutation p-values.
## [bumphunterEngine] cutoff: 0.238
## [bumphunterEngine] Finding regions.
## Warning in regionFinder(x = beta, chr = chr, pos = pos, cluster =
## cluster, : NAs found and removed. ind changed.
## [bumphunterEngine] Found 9153 bumps.
## [bumphunterEngine] Computing regions for each permutation.
## Warning in FUN(newX[, i], ...): NAs found and removed. ind changed.

## Warning in FUN(newX[, i], ...): NAs found and removed. ind changed.

## Warning in FUN(newX[, i], ...): NAs found and removed. ind changed.
## [bumphunterEngine] Estimating p-values and FWER.
```

Here in this example data-set there is very little difference. However when testing this very function in a dataset of 1,200 EPIC arrays, the bigmelon version I have written uses very little memory (it depends on the number of bootstraps you want to do) the original bumphunter used in excess for 40Gb of memory, however this function takes a considerably longer time than just simply extracting the values and feeding it to the original function. It is likely that the function could be further optimised (through parallelisation) but in the interest of keeping this short we will leave it here.

```r
# Closing and deleting gds file for this example
closefn.gds(gfile)
unlink('melon.gds')
```

```r
sessionInfo()
```

```
## R version 3.4.3 (2017-11-30)
## Platform: x86_64-pc-linux-gnu (64-bit)
## Running under: Ubuntu 14.04.5 LTS
##
## Matrix products: default
## BLAS: /usr/lib/libblas/libblas.so.3.0
## LAPACK: /usr/lib/lapack/liblapack.so.3.0
##
## locale:
##  [1] LC_CTYPE=en_GB.UTF-8       LC_NUMERIC=C
##  [3] LC_TIME=en_GB.UTF-8        LC_COLLATE=en_GB.UTF-8
##  [5] LC_MONETARY=en_GB.UTF-8    LC_MESSAGES=en_GB.UTF-8
##  [7] LC_PAPER=en_GB.UTF-8       LC_NAME=C
##  [9] LC_ADDRESS=C               LC_TELEPHONE=C
## [11] LC_MEASUREMENT=en_GB.UTF-8 LC_IDENTIFICATION=C
```

```
##
## attached base packages:
## [1] stats4    parallel  stats     graphics  grDevices utils     datasets
## [8] methods   base
##
## other attached packages:
##  [1] doRNG_1.6.6
##  [2] rngtools_1.2.4
##  [3] pkgmaker_0.22
##  [4] registry_0.5
##  [5] minfiData_0.24.0
##  [6] IlluminaHumanMethylation450kmanifest_0.4.0
##  [7] microbenchmark_1.4-3
##  [8] bigmelon_1.5.7
##  [9] gdsfmt_1.14.1
## [10] wateRmelon_1.25.1
## [11] illuminaio_0.20.0
## [12] IlluminaHumanMethylation450kanno.ilmn12.hg19_0.6.0
## [13] ROC_1.54.0
## [14] lumi_2.30.0
## [15] methylumi_2.24.1
## [16] minfi_1.24.0
## [17] bumphunter_1.20.0
## [18] locfit_1.5-9.1
## [19] iterators_1.0.9
## [20] foreach_1.4.4
## [21] Biostrings_2.46.0
## [22] XVector_0.18.0
## [23] SummarizedExperiment_1.8.1
## [24] DelayedArray_0.4.1
## [25] FDb.InfiniumMethylation.hg19_2.2.0
## [26] org.Hs.eg.db_3.5.0
## [27] TxDb.Hsapiens.UCSC.hg19.knownGene_3.2.2
## [28] GenomicFeatures_1.30.0
## [29] AnnotationDbi_1.40.0
## [30] GenomicRanges_1.30.1
## [31] GenomeInfoDb_1.14.0
## [32] IRanges_2.12.0
## [33] S4Vectors_0.16.0
## [34] ggplot2_2.2.1
## [35] reshape2_1.4.3
## [36] scales_0.5.0
## [37] matrixStats_0.53.0
## [38] limma_3.34.8
## [39] Biobase_2.38.0
## [40] BiocGenerics_0.24.0
##
## loaded via a namespace (and not attached):
##  [1] TH.data_1.0-8            colorspace_1.3-2
##  [3] siggenes_1.52.0          mclust_5.4
##  [5] rprojroot_1.3-2          base64_2.0
##  [7] affyio_1.48.0            bit64_0.9-7
##  [9] mvtnorm_1.0-6            xml2_1.2.0
## [11] codetools_0.2-15         splines_3.4.3
```

```
## [13] knitr_1.18               Rsamtools_1.30.0
## [15] annotate_1.56.1          readr_1.1.1
## [17] compiler_3.4.3           httr_1.3.1
## [19] backports_1.1.2          assertthat_0.2.0
## [21] Matrix_1.2-12            lazyeval_0.2.1
## [23] htmltools_0.3.6          prettyunits_1.0.2
## [25] tools_3.4.3              bindrcpp_0.2
## [27] gtable_0.2.0             glue_1.2.0
## [29] GenomeInfoDbData_1.0.0   affy_1.56.0
## [31] dplyr_0.7.4              Rcpp_0.12.15
## [33] multtest_2.34.0          preprocessCore_1.40.0
## [35] nlme_3.1-131             rtracklayer_1.38.2
## [37] stringr_1.2.0            XML_3.98-1.9
## [39] beanplot_1.2             nleqslv_3.3.1
## [41] zoo_1.8-1                zlibbioc_1.24.0
## [43] MASS_7.3-48              BiocInstaller_1.28.0
## [45] hms_0.4.0                sandwich_2.4-0
## [47] GEOquery_2.46.14         RColorBrewer_1.1-2
## [49] yaml_2.1.16              memoise_1.1.0
## [51] biomaRt_2.34.1           reshape_0.8.7
## [53] stringi_1.1.6            RSQLite_2.0
## [55] genefilter_1.60.0        RMySQL_0.10.13
## [57] BiocParallel_1.12.0      rlang_0.1.6
## [59] pkgconfig_2.0.1          bitops_1.0-6
## [61] nor1mix_1.2-3            evaluate_0.10.1
## [63] lattice_0.20-35          purrr_0.2.4
## [65] bindr_0.1                GenomicAlignments_1.14.1
## [67] bit_1.1-12               plyr_1.8.4
## [69] magrittr_1.5             R6_2.2.2
## [71] multcomp_1.4-8           DBI_0.7
## [73] pillar_1.1.0             mgcv_1.8-23
## [75] survival_2.41-3          RCurl_1.95-4.10
## [77] tibble_1.4.2             KernSmooth_2.23-15
## [79] rmarkdown_1.8            progress_1.1.2
## [81] grid_3.4.3               data.table_1.10.4-3
## [83] blob_1.1.0               digest_0.6.14
## [85] xtable_1.8-2             tidyr_0.8.0
## [87] openssl_0.9.9            munsell_0.4.3
## [89] quadprog_1.5-5
```
