# Supplementary material for: Bigmelon: tools for analysing large DNA methylation datasets
Source: Bioinformatics. 2018 Aug 23;35(6):981–6. doi: 10.1093/bioinformatics/bty713 (PMC6419913; doi:10.1093/bioinformatics/bty713)
Supplement: Supplementary Data [file bty713_supp.zip › bty713-suppl_data/bigmelon_Suppl_Figures.pdf]

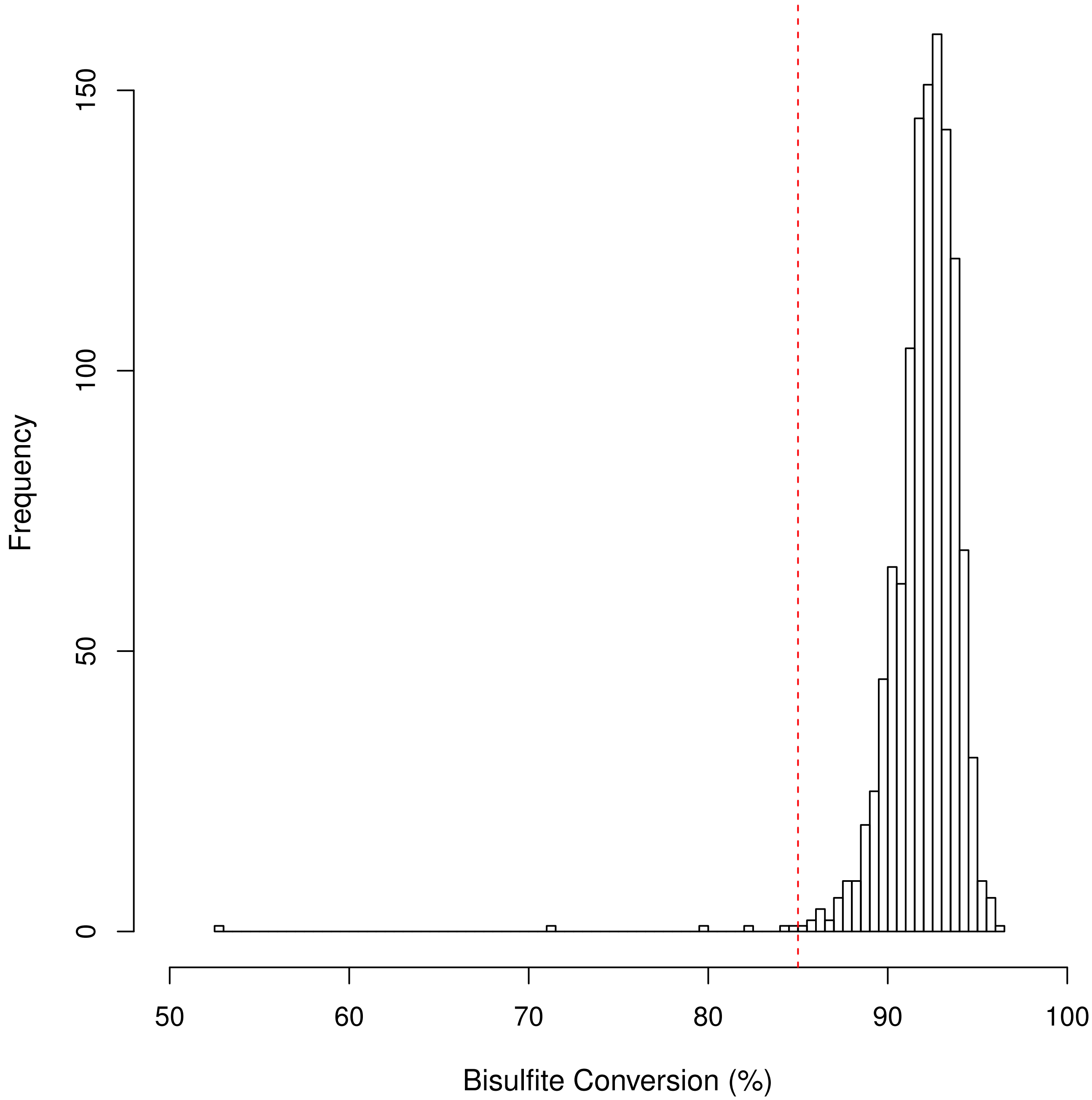

Supplementary Figure 1: Histogram of bisulfite conversion percentages from *Understanding Society:* UK Household dataset as estimated by the `bscon` function in wateRmelon. Conservative threshold of 85% represented with red-dashed line is used to filter out low-quality samples.

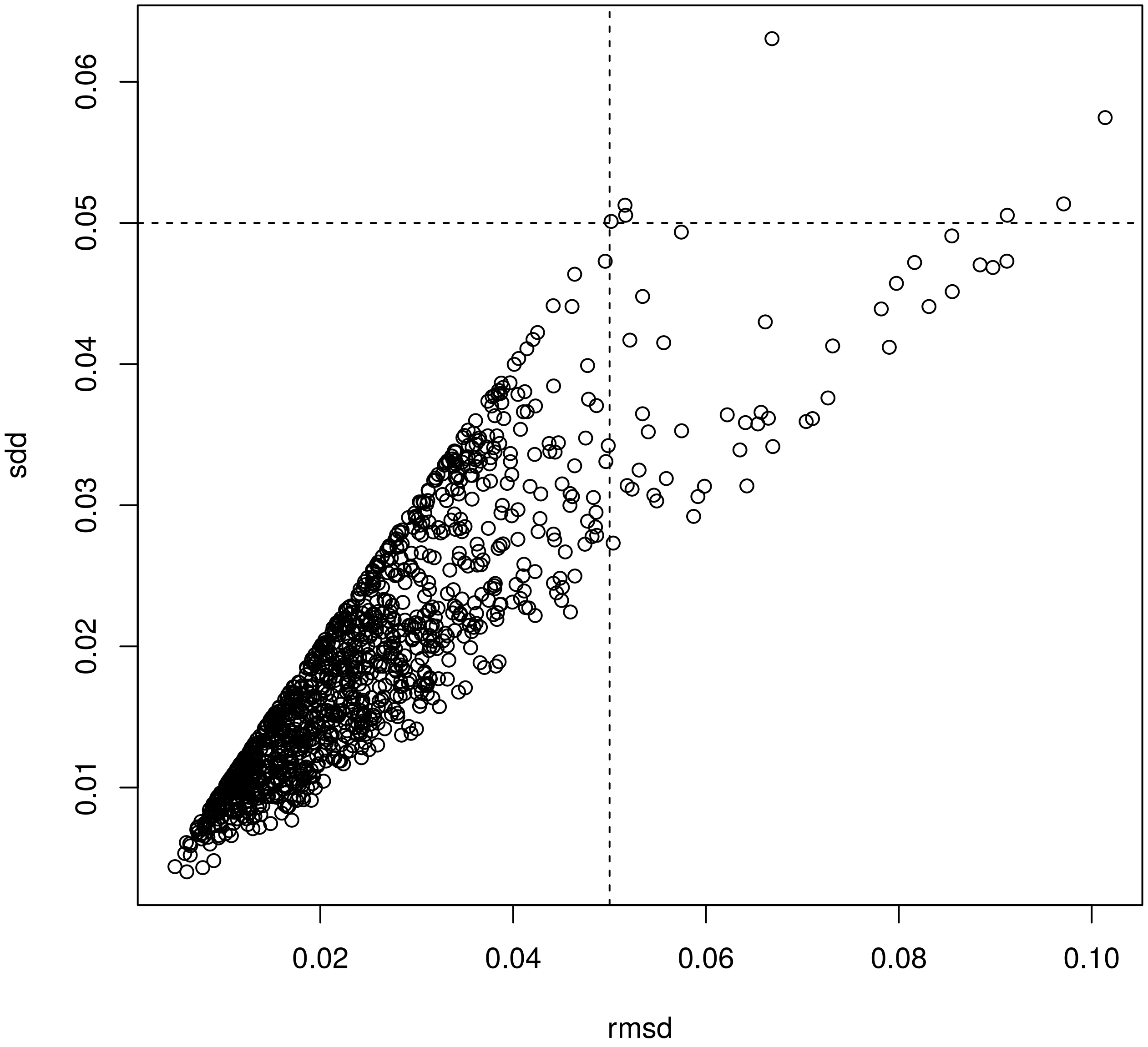

Supplementary Figure 2: Differences between `dasen` normalised and raw β values from *Understanding Society:* UK Household dataset as calculated by the `qual` function in wateRmelon package. Samples above thresholds of 0.05 (dashed lines) Root Mean Square Difference (rmsd) or Standard Deviation of Difference (sdd) were excluded from further analysis as they represent data that have undergone the most change during normalisation. Each data point represents a single sample.

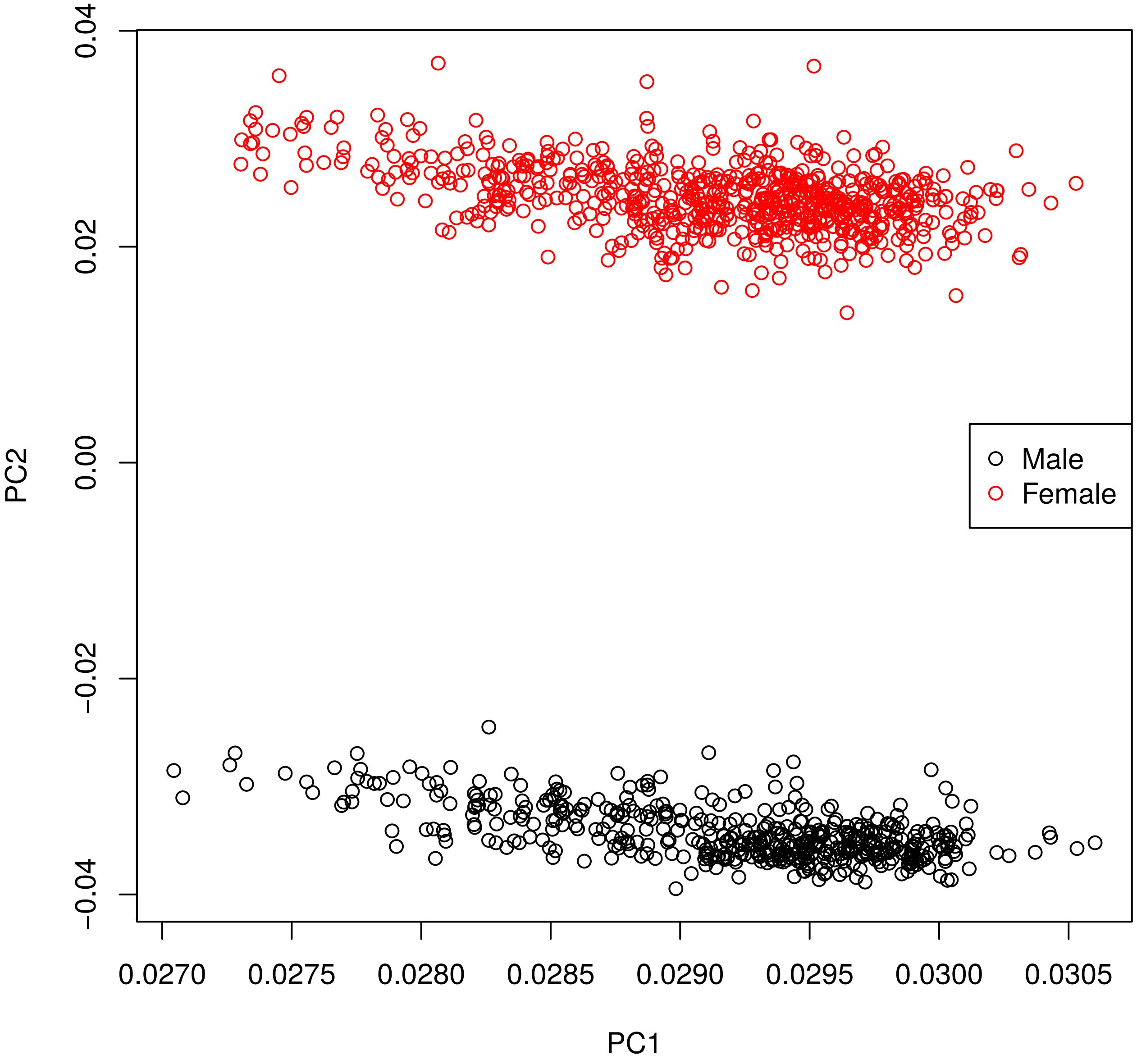

Supplementary Figure 3: Scatter plot of loading vectors from Principal Components 1 and 2 from *Understanding Society:* UK Household dataset calculated from a random 1% of data. Data-points correspond to individual samples and coloured by annotated sex.

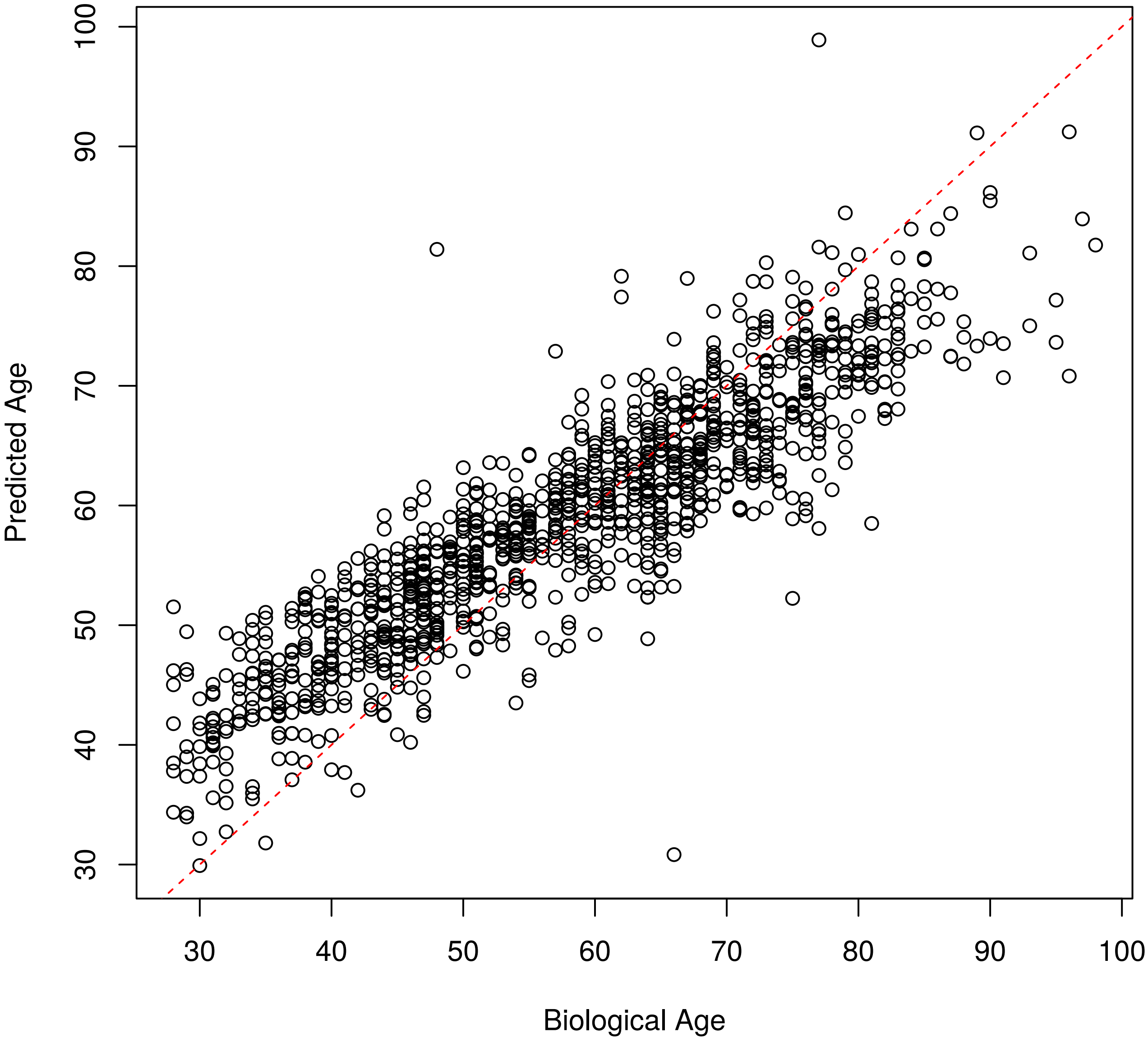

Supplementary Figure 4: Example of age prediction on the *Understanding Society:* UK Household dataset, calculated by the `agep` function in the wateRmelon package, red dashed lines represented a perfect fit between biological and predicted age.

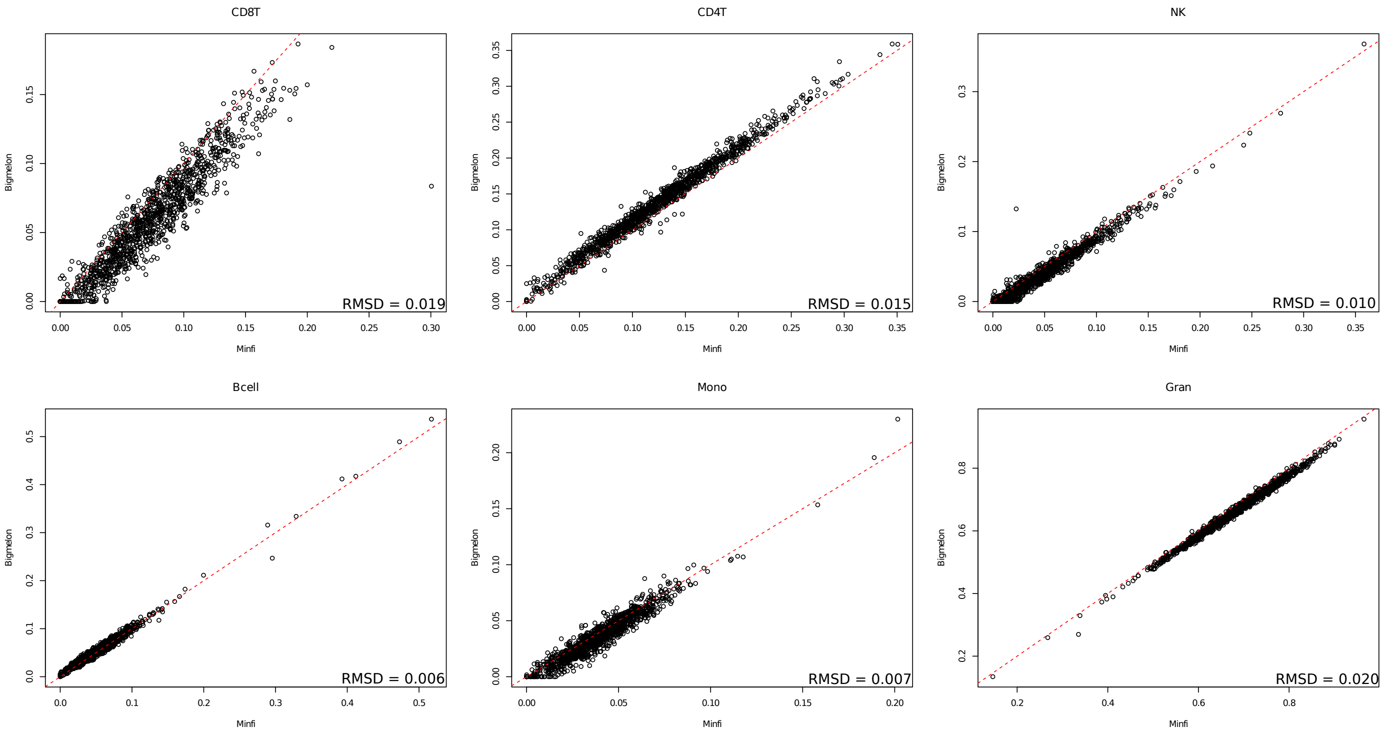

Supplementary Figure 5: Comparison between bigmelon and minfi methods of cell-type composition estimations. minfi cell-type compositions estimated using the estimateCellCounts function and bigmelon cell-type composition estimations determined using estimateCellCounts.gds function. estimateCellCounts.gds functions similar to the minfi version however differs only when normalising the biological data with the reference dataset, by normalising reference dataset using the biological dataset quantiles rather than normalising the two datasets together. Root mean square difference was calculated for each predicted cell-type to show overall good precision despite differing methodologies.

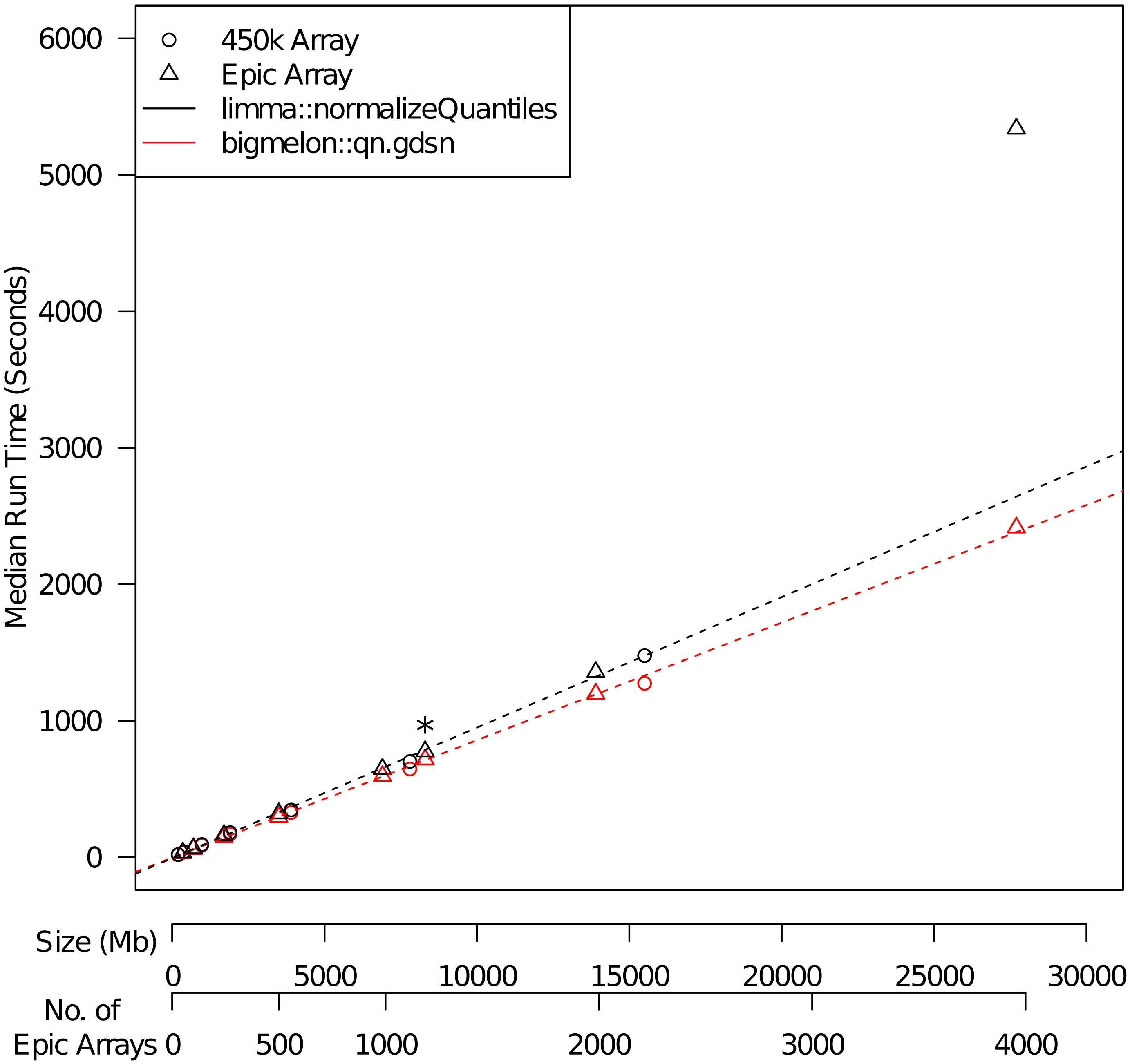

Supplementary Figure 6: Median run time of quantile normalisation methods of bigmelon and limma on increasing numbers of microarrays. $*$ denotes the size (in Gb) of roughly 1200 HumanMethylationEPIC arrays. Analysis was performed on a workstation with 128Gb of memory. Speeds remain comparable up until 25Gb where limma::normalizeQuantiles function runs out of memory, on workstations with less memory the breaking point is much less.
