# Supplementary material for: Bigmelon: tools for analysing large DNA methylation datasets
Source: Bioinformatics. 2018 Aug 23;35(6):981–6. doi: 10.1093/bioinformatics/bty713 (PMC6419913; doi:10.1093/bioinformatics/bty713)
Supplement: Supplementary Data [file bty713_supp.zip › bty713-suppl_data/Suppl_Materials_1_Concurrent_Mem_usage.doc]

*Suppl. Materials*

**Concurrent memory usage:**

Given how standard work-flows currently exist it is necessary to parse the raw idat files in pairs and convert them into meaningful values. This can consume a lot of memory before any meaningful analysis has even taken place. For example: Parsing a single pair of idat files (450k) within minfi requires a minimum 76 MB of memory with each additional pair of idat files contributing an additional 4.8 MB to the total object size (of the RGChannelSet, not including detection p-values or beadcounts). Typical work-flow dictates that data is further processed into a MethylSet (either through processing or not) which then additionally requires at least 60 MB of memory plus 7.4 MB per sample. Lastly, analysis is often performed on Beta values which are the ratio between Methylated and Unmethylated values. Which in total requires 30 MB plus 3.7 MB per sample.

The consumption of memory can be largely avoided through garbage collection and deleting unneeded memory objects after processing. However assuming that after going through a pipeline a resultant beta matrix containing 1,000 samples there is approximately (3.7GB and 6.6GB of memory are being used to store the beta matrix alone for 450k and EPIC array respectively.) These estimations in data-sizes are estimates that do not include other encoded data within the idats such as detection-p values and bead counts. Which also influence the total memory requirement for reading in data.

Linear functions of memory usage for minfi given increasing n for 450K and EPIC micro-arrays. Generalised object sizes determined using object.size() function on increasing sizes of objects from minfi.

**N.B. This is estimated using RGChannelSet instead of RGChannelSetExtended which contain information that can be used to compute detection-p values and bead counts and take up more memory.**

**450k::**

M_RG-450K_	= n4.8 + 76

M_MSet-450K_ = n7.4 + 60

M_Beta-450K_ = n3.7 + 30

M_total-450K_ = M_RG-450K_ + M_MSet-450K_ + M_beta-450K_

M_total-450K_ = n4.8 + n7.4 + n3.7 + 166

**EPIC::**

M_RG-EPIC_	= n8 + 126.7

M_MSet-EPIC_ = n13.3 + 105.7

M_Beta-EPIC_ = n6.6 + 52.9

M_total-EPIC_ = M_RG-EPIC_ + M_MSet-EPIC_+ M_Beta-EPIC_

*M_total-EPIC_ = n8 + n13.3 + n6.6 + 285.3*
